# Early Dose-Related Cardiorenal Effects of Cisplatin: Integrated Biochemical, Molecular and Histopathological Evaluation in an Experimental Rat Model

**DOI:** 10.3390/biomedicines14071490

**Published:** 2026-06-30

**Authors:** Gülsüm Abuşoğlu, Melek Altunkaya, Mehmet Burak Ateş, Ayşegül Bulut, Bahadır Öztürk

**Affiliations:** 1 Medical Laboratory Techniques Program, Vocational School of Health Services, Selcuk University, Konya 42130, Turkey; melek.batakci@selcuk.edu.tr; 2 Department of Pathology, Faculty of Veterinary, Selcuk University, Konya 42130, Turkey; mehmetburakates@selcuk.edu.tr (M.B.A.); vetaysegulbulut@gmail.com (A.B.); 3 Department of Biochemistry, Faculty of Medicine, Selcuk University, Konya 42130, Turkey; ozturkbhdr@hotmail.com

**Keywords:** cisplatin, cardiorenal toxicity, dose–response, oxidative stress, histopathology, experimental toxicology

## Abstract

**Background/Objectives**: Cisplatin (CP) is a widely used chemotherapeutic agent; however, its dose-dependent effects on different tissues are not fully understood. This study aimed to investigate the early dose-related cardiorenal toxicity of CP at biochemical, molecular, and histopathological levels. **Methods**: Male Wistar albino rats were divided into four groups: Control, 5 mg/kg CP, 7.5 mg/kg CP, and 12 mg/kg CP. Cardiac and renal tissues were collected three days after administration. Oxidative stress and inflammatory parameters were analyzed using ELISA. Histopathological evaluation and semi-quantitative scoring were performed on tissue sections. Apoptosis-related proteins were assessed using Western blotting and immunohistochemistry. **Results**: In both renal and cardiac tissues, LPO, MDA, and SOD levels showed significant dose-dependent changes, whereas inflammatory parameters did not differ significantly among the groups. Although Bax and Bcl-2 proteins displayed significant dose-dependent variations in both tissues at the protein level, immunohistochemical analyses showed no notable differences in cardiac tissue. **Conclusions**: Acute cisplatin exposure produced dose-related biochemical, molecular, and histopathological alterations in both cardiac and renal tissues. Oxidative stress-related changes were more prominent than cytokine-mediated inflammatory responses during the acute experimental period. Within the conditions of this acute model, lower cisplatin doses were associated with less pronounced tissue alterations.

## 1. Introduction

Cis-diamminedichloroplatinum (cisplatin, CP) is a widely used chemotherapeutic agent against a variety of tumors, including testis, bladder, ovary, head and neck, cervix, lung, and endometrium [1]. Nevertheless, its full clinical application is limited due to its adverse effects on the inner ear, peripheral nerves, heart, kidneys, and testes [2,3]. In individuals undergoing CP treatment, physiological homeostasis is disrupted [4].

Oxidative stress and inflammation play significant roles in this disruption, both of which are associated with CP-induced toxicity [5]. The dose-limiting side effects of CP include nephrotoxicity, neurotoxicity, and ototoxicity [6]. Among these, sensory neuropathy and kidney injury are two of the most common adverse effects that restrict CP therapy. Regarding kidney injury, this antitumor agent has been reported to cause tubular damage and tubular dysfunction accompanied by the loss of sodium, potassium, and magnesium, and to lead to a dose-dependent decrease in glomerular filtration rate [7]. However, the most frequently reported adverse effect of CP is nephrotoxicity, which has been shown to vary with dose and cumulative exposure [8,9]. CP-induced nephrotoxicity is dose-dependent and involves necrosis, apoptosis, and necroptosis of renal cells [10,11]. In vitro studies have demonstrated that necrotic cell death results from high concentrations of CP, whereas apoptosis is triggered by lower concentrations [10,12]. Moreover, several recent studies have revealed a substantial increase in the risk of cardiovascular events among cancer patients treated with CP, indicating that CP-induced cardiovascular toxicity has become a growing concern. Chronic CP treatment has been suggested to trigger cardiac and vascular toxicity in a dose-dependent manner [2]. Following CP administration, cell death can be initiated through apoptotic pathways: the extrinsic, intrinsic (mitochondrial), and endoplasmic reticulum (ER) stress-mediated pathways. CP activates caspases-3, -8, and -9, and triggers the translocation of Bax to the mitochondria, leading to the release of cytochrome c, apoptosis-inducing factor (AIF), and endonuclease G [11,13,14]. Inhibition of caspase-3 and caspase-9 suppresses CP-induced cell death [15], while mice deficient in Bax treated with CP exhibit reduced cytochrome c release [13]. This finding highlights the crucial roles of both the extrinsic and intrinsic apoptotic pathways in CP-induced cytotoxicity.

The dose-dependence of CP-induced toxicity suggests that the extent of toxicity may vary among different tissues. However, both in vivo and in vitro studies remain insufficient to clearly determine the dose ranges at which varying levels of toxicity occur. Experimental animal models, particularly rat models, are widely used in toxicological research due to their physiological and biochemical similarities to humans and their suitability for investigating organ-specific responses. We hypothesized that CP induces dose-dependent and tissue-specific alterations in cardiac and renal tissues, with higher doses leading to more pronounced biochemical, molecular, and histopathological changes. This study aimed to characterize early dose-dependent cardiorenal toxicity of cisplatin using an integrated multi-level approach, with particular emphasis on identifying tissue-specific early toxic responses and discrepancies between biochemical, molecular, and histopathological findings.

## 2. Materials and Methods

### 2.1. Study Design and Animal Experimentation

In this study, the rats were sourced from the Selçuk University Experimental Medicine Application and Research Center (Konya, Türkiye). Wistar albino rats (male; 8–12 weeks) were used. Animals were maintained under standard conditions with ad libitum access to food and water. The sample size was determined based on previous experimental cisplatin toxicity studies reporting comparable biochemical, molecular, and histopathological endpoints together with similar animal group sizes [2,5]. The environmental conditions, including light, temperature, and humidity, were maintained under controlled settings (12/12 h light/dark cycle, 22 ± 2 °C, 55 ± 5% humidity). Animals were randomly allocated to experimental groups using a simple randomization approach. All procedures were conducted at the same time of day, and animals were housed under standardized environmental conditions to minimize potential confounding effects. Animals were allowed to acclimatize to the laboratory environment for 7 days prior to the experimental procedures. Control group (*n* = 8): received a single intraperitoneal (i.p.) dose of 1 mL 0.9% isotonic saline; CP 5 mg/kg group (*n* = 10): received a single i.p. dose of 5 mg/kg CP [16]; CP 7.5 mg/kg group (*n* = 10): received a single i.p. dose of 7.5 mg/kg CP [17]; CP 12 mg/kg group (*n* = 10): received a single i.p. dose of 12 mg/kg CP [18]. CP (50 mg/100 mL IV, Koçak Farma, İstanbul, Türkiye) was obtained commercially and administered intraperitoneally. Three days after CP administration, animals were anesthetized and heart and kidney tissues were collected. General anesthesia was induced using Ketalar (ketamine hydrochloride; Pfizer Inc., New York, NY, USA; 90 mg/kg, i.p.) and Rompun (xylazine hydrochloride; Bayer AG, Leverkusen, Germany; 10 mg/kg, i.p.). Depth of anesthesia was confirmed by assessment of muscle tone, palpebral reflex, and response to pinching. Animals were euthanized by cervical dislocation under deep anesthesia. All procedures were performed in accordance with ethical guidelines to minimize animal suffering. Animals were monitored daily for general health status and signs of distress throughout the experimental period. No unexpected adverse events were observed. No predefined humane endpoints were established, and all animals were sacrificed at the predetermined experimental endpoint. The study was conducted in accordance with the Guide for the Care and Use of Laboratory Animals. Ethical approval was granted by the Animal Experiments Ethics Committee of the Selçuk University Experimental Medicine Application and Research Center (decision number: 2024/15; date: 29 February 2024).

### 2.2. Preparation of Tissue Homogenates

For the analysis of oxidative stress and inflammation parameters, tissue homogenates were prepared using 0.01 M phosphate buffer (pH 7.4). Briefly, tissues were homogenized on ice in the presence of the buffer solution. Following homogenization, the samples were centrifuged at 3000× *g* for 10 min, and the resulting supernatants were aliquoted and stored at −80 °C until the day of analysis.

### 2.3. Protein Analysis from Tissues

Prior to Western blot analysis, tissue lysates were prepared on ice using RIPA lysis buffer 1 g tissue/10 mL RIPA (Cat no: E-BC-R327, Elabscience, Houston, TX, USA) supplemented with 100 mM Na_3_VO_4_ (Cat no: E-BC-R250, Elabscience) and 100 mM PMSF (Cat no: E-BC-R287, Elabscience). The lysates were centrifuged at 12,000× *g* for 10 min at +4 °C, and the resulting supernatants were collected for total protein quantification. Total protein levels were defined spectrophotometrically at 562 nm using the Bicinchoninic Acid (BCA; Cat no: ZH394546, Thermo Scientific, Waltham, MA, USA) method. In this assay, bovine serum albumin (BSA, 0–2000 µg/mL) was used as a standard, and protein concentrations of the samples were calculated based on the calibration curve. All procedures were performed in accordance with the manufacturer’s protocol.

### 2.4. Biochemical Studies, ELISA Analyses and Serological Tests

For the evaluation of oxidative stress and inflammation in tissue samples, the levels of malondialdehyde (MDA, Cat no: E0156Ra, BT LAB/Bioassay Technology Laboratory, Shanghai Korain Biotech Co., Ltd., Shanghai, China), lipid peroxidase (LPO, Cat no: E0285Ra, BT LAB), superoxide dismutase (SOD, Cat no: E0168Ra, BT LAB), and 4-hydroxynonenal (4-HNE, Cat no: MBS1609751, MyBiosource, San Diego, CA, USA), as well as the inflammatory cytokines interleukin-6 (IL-6, Cat no: E0135Ra, BT LAB), interleukin-10 (IL-10, Cat no: E0108Ra, BT LAB), interleukin-1β (IL-1β, Cat no: E0119Ra, BT LAB), and tumor necrosis factor-α (TNF-α, Cat no: E0764Ra, BT LAB), were measured using enzyme-linked immunosorbent assay (ELISA) kits. All measurements were performed spectrophotometrically at 450 nm, and all procedures followed the protocols provided by the respective manufacturers. Serological analyses were performed using plasma samples obtained from rats to assess cardiac and renal function. Renal function parameters included urea and creatinine levels, while cardiac function was evaluated by measuring CK-MB and Troponin T levels. Biochemical and hormone analyses were carried out using Roche Cobas analyzers (Roche Diagnostics GmbH, Mannheim, Germany).

### 2.5. Apoptotic Protein Analysis, Western Blot

Protein levels of the tissue samples were equalized and adjusted to 15 µg/mL using sample dilution buffer, followed by incubation at 90 °C for 5 min. The samples were then loaded into the wells of a Mini-PROTEAN Tetra Cell Gel (Mini-PROTEAN Tetra Cell electrophoresis system (Bio-Rad Laboratories, Hercules, CA, USA) and separated by electrophoresis at 30 mA for 120 min. The resolved protein bands were subsequently transferred onto a PVDF membrane at 25 mA for 30 min. Following transfer, the membranes were blocked at room temperature (RT) for 1 h with 5% non-fat dry milk and incubated overnight at 4 °C with the following primary antibodies: anti-Bax (1:1000; AB32503, Abcam, Cambridge, UK), anti-Bcl-2 (1:1000; AB194583, Abcam). GAPDH (1:1000; E-AB-40337, Elabscience) was used as an internal control. After incubation, the membranes were washed three times for 15 min each at room temperature and then incubated for 1 h with an HRP-conjugated goat anti-rabbit IgG secondary antibody (1:5000; Cat no: E-AB-1003, Elabscience). Subsequent to another three 15 min washing steps, the membranes were incubated with Opti-4CN substrate reagent (Cat No: 1708225, Bio-Rad Laboratories, Hercules, CA, USA) for 10 min in the dark at RT. Protein bands were visualized and quantified using a Gel Doc XR+ imaging system (Bio-Rad Laboratories, Hercules, CA, USA), and band intensities were analyzed using the corresponding software.

### 2.6. Histopathological Analyses

Following necropsy, the kidney and heart tissues of the rats were dissected in accordance with standard procedures. For histopathological and immunohistochemical examinations, the tissues were fixed in 10% formaldehyde solution for 24 h. After fixation, the samples were trimmed to appropriate sizes and placed in tissue processing cassettes. The tissues were then washed under running water for 12 h before being processed in a routine tissue processor (Leica TP 1020, Leica Biosystems, Nussloch, Germany). Subsequently, the tissues were embedded in paraffin, and 5 µm-thick sections were obtained using a microtome (Leica RM 2125RT, Leica Biosystems) for both hematoxylin–eosin (H&E) and immunohistochemical staining. Routine histopathological evaluations were performed according to the hematoxylin–eosin staining method described by Luna [19]. All slides were examined under a light microscope (Olympus BX51, Olympus, Tokyo, Japan). To ensure comparability between groups in histopathological and immunohistochemical examinations, sections were prepared from the same region of the kidney and heart tissues of 8 randomly selected animals per group. Microscopic evaluation of the kidney sections included the assessment of hydropic degeneration, necrosis, tubular dilatation, glomerular atrophy, hyaline casts, interstitial mononuclear cell infiltrate (IMCI), interstitial edema, perivascular edema, congestion, and hemorrhage. These parameters were scored semi-quantitatively on a scale of 0 to 4 (0: no lesion, 1: mild, 2: moderate, 3: severe, 4: very severe) [20,21]. Cardiac sections were evaluated for degeneration, necrosis, congestion, hemorrhage, interstitial edema, and mononuclear cell infiltrate (MCI) scored on a scale of 0 to 3 (0: no lesion, 1: mild, 2: moderate, 3: severe) [22,23]. All sections of kidney and heart tissues were evaluated, and each histopathological parameter was examined and scored separately. Examinations were performed at magnifications between 10× and 40×, depending on the characteristics of the lesion being evaluated. Histopathological evaluations were performed jointly by two experienced veterinary pathologists, unaware of the groups, and scores were assigned to each parameter based on a joint decision. Prior to evaluation, the preparations were coded by a researcher who was not involved in the pathological examination process. Representative photomicrographs from both kidney and heart tissues were captured using an Olympus EP50 camera system (Evident Corporation, Tokyo, Japan).

### 2.7. Immunohistochemical Analyses

For immunohistochemical (IHC) staining, 5 µm-thick sections of kidney and heart tissues were mounted on adhesive slides. It was performed using a Leica Bond-Max automated immunohistochemistry system in accordance with the Bond™ Polymer Refine Detection Kit protocol (Leica DS9800, Leica Biosystems), which includes the following sequential steps: Peroxidase Block, Protein Block, Post-Primary, Polymer, DAB, and Hematoxylin. All sections were deparaffinized using a heating and dewax solution (Bond™, Leica AR9222, Leica Biosystems) and subsequently rehydrated through a graded series of alcohols (100–70%) (Sigma). During the staining process, to remove residual reagents or following marker application, all sections were washed at least three times with either wash buffer (Bond™, Leica AR9590, Leica Biosystems) and/or distilled water. Heat-induced epitope retrieval (HIER 1) was performed using Citrate Buffer (pH 6.0; Leica AR9961, Leica Biosystems). To prevent nonspecific binding, peroxidase and protein blocking were applied for 30 min each. The kidney sections were then incubated at RT for 30 min with the following primary antibodies: anti-Bax antibody (Abcam (Cambridge, UK), ab32503; 1:250) and anti-Bcl-2 antibody (Elabscience, E-AB-60012; 1:100). Heart sections were incubated at RT for 45 min with anti-Bax antibody (Abcam, ab32503; 1:200) and anti-Bcl-2 antibody (Elabscience, E-AB-60012; 1:50). After primary antibody incubation, Post-Primary and Polymer reagents were applied for 10 min each. All sections were treated with 3,3′-diaminobenzidine (DAB) chromogen for 3 min and rinsed with distilled water. Counterstaining was performed using Mayer’s hematoxylin for 2 min, followed by dehydration and mounting with Entellan (Merck, Darmstadt, Germany). The stained slides were examined under a light microscope (Olympus BX51, Olympus, Tokyo, Japan). For each slide, five random fields were evaluated at 40× magnification. The intensity and distribution of Bax and Bcl-2 immunoreactivity were assessed using the Allred scoring method [24]. Immunohistochemical stainings were evaluated by two experienced veterinary pathologists unaware of the groups. The preparations were coded prior to evaluation, and group information was not shared with the pathologists during the examination. Scoring for Bax and Bcl-2 immunoreactivity was determined by consensus based on the combined evaluation of the preparations. Representative photomicrographs were captured using an Olympus EP50 imaging system (Evident Corporation).

### 2.8. Statistical Analyses

Data analysis was carried out using Microsoft Excel and IBM SPSS Statistics software (Version 21). The normality of data distribution was evaluated through the Kolmogorov–Smirnov test. For datasets demonstrating normal distribution, a one-way analysis of variance (ANOVA) was applied, followed by Tukey’s post hoc Honestly Significant Difference (HSD) test. All results were presented as mean ± standard deviation (SD), and statistical significance was accepted at a level of *p* < 0.05.

## 3. Results

### 3.1. Evaluation of Heart and Kidney Function Markers

**Figure 1:**
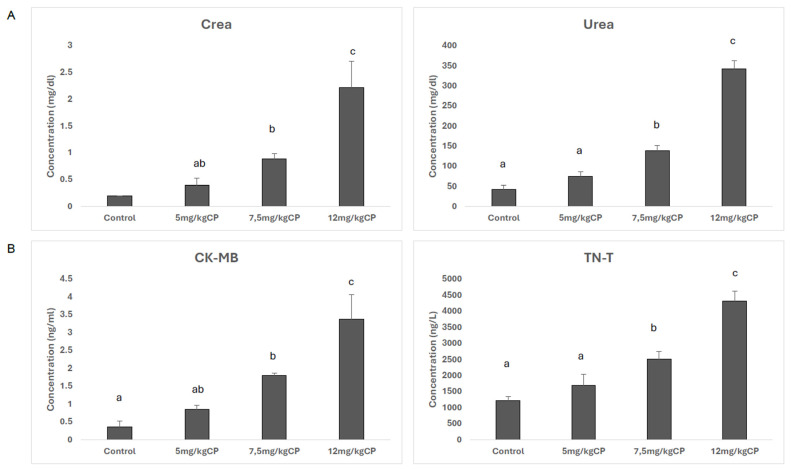
Serological analyses of plasma samples obtained from rats treated with different doses of CP were evaluated using routine biochemistry and hormone analyzers. (**A**) Creatinine and urea levels for kidney tissue; (**B**) troponin T and CK-MB levels for heart tissue. Different letters in the same column are statistically significant according to one-way ANOVA and post hoc Tukey’s HSD test. CP: Cisplatin.

Compared with the control group, urea and creatinine levels, as renal function markers, showed a significant increase. Although the 5 mg/kg group (*p* = 0.048 and *p* = 0.0802, respectively) did not differ from the control group, both urea and creatinine levels were significantly elevated in the 7.5 mg/kg group (*p* < 0.001 and *p* = 0.002, for urea and creatinine, respectively) and in the 12 mg/kg group (*p* < 0.001 and *p* < 0.001, for urea and creatinine, respectively) (Figure 1A). Similarly, in the assessment of cardiac function, both troponin T (TnT) and CK-MB levels increased significantly with increasing CP dose (*p* < 0.001 and *p* < 0.001, for CK-MB and TnT, respectively), whereas no significant difference compared with the control group was observed in the 5 mg/kg group (Figure 1B).

### 3.2. Effect of CP on Oxidative Stress and Inflammation in Tissues

**Figure 2:**
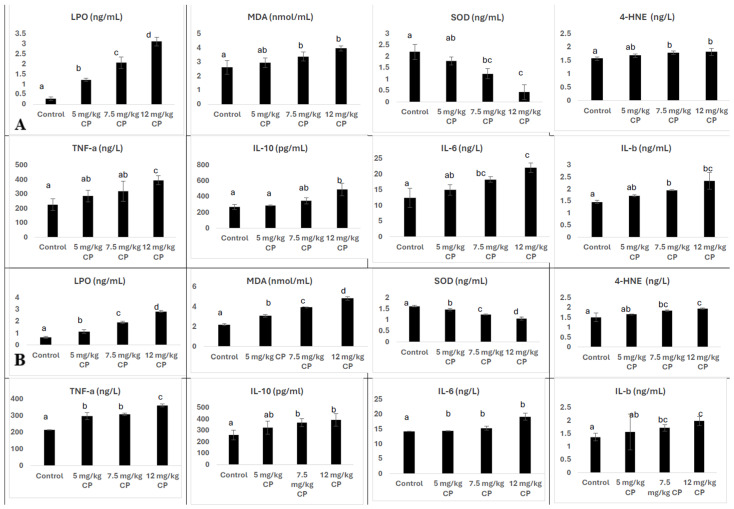
Dose-dependent effects of CP on oxidative stress and inflammatory responses in kidney and heart tissues. The dose-dependent changes in LPO, MDA, SOD, and 4-HNE levels, as well as TNF-α, IL-10, IL-6, and IL-β concentrations, in kidney (**A**) and heart (**B**) tissues following administration of different doses of CP. Different letters in the same column are statistically significant according to one-way ANOVA and post hoc Tukey’s HSD test. CP: Cisplatin.

The levels of selected oxidative stress markers, including MDA, SOD, LPO, and 4-HNE, were determined by ELISA. According to the results, selected oxidative stress markers (MDA (*p* = 0.03) and LPO (*p* < 0.001)) showed a statistically significant increase in kidney and heart tissues with increasing doses of CP, whereas SOD (*p* = 0.042) levels demonstrated a significant decrease. While MDA and LPO levels increased significantly, 4-HNE levels did not show a statistically significant change, suggesting a differential response among oxidative stress markers (*p* = 0.189). (Figure 2A,B). Among the inflammation parameters, TNF-α levels were similar in the 5 (*p* = 0.07) and 7.5 mg/kg (*p* = 0.053) groups but showed a significant increase in the 12 mg/kg group (*p* = 0.04). IL-6 (*p* = 0.296), IL-10 (*p* = 0.167), and IL-1β (*p* = 0.278) levels differed from the control group in CP-treated animals; however, these cytokines did not show a clear progressive dose-dependent pattern across CP doses (Figure 2A,B). Although certain cytokines showed mild elevations, most inflammatory markers did not demonstrate a strong progressive dose-dependent activation pattern during the acute experimental period.

### 3.3. Effects of CP on Apoptotic Protein Levels

**Figure 3:**
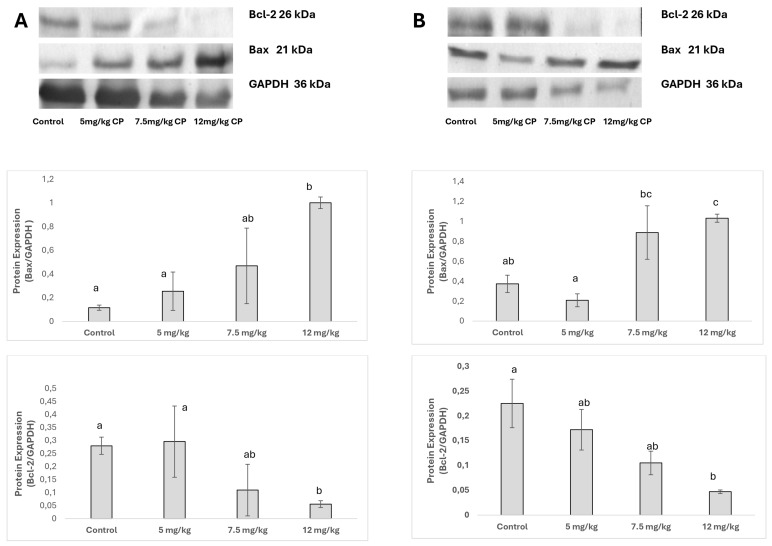
The effects of different doses of CP on anti-apoptotic and pro-apoptotic protein levels in heart and kidney tissues. (**A**) Graphical and blot-based representation of Bax, Bcl-2, and housekeeping protein (GAPDH) levels in kidney tissue. (**B**) Graphical and blot-based representation of Bax, Bcl-2, and housekeeping protein (GAPDH) levels in heart tissue. Different letters in the same column are statistically significant according to one-way ANOVA and post hoc Tukey’s HSD test. CP: Cisplatin.

The expression levels of apoptotic pathway proteins Bax and Bcl-2 were analyzed in rat heart and kidney tissues treated with different doses of CP using Western blot analysis. Representative blot images and the corresponding densitometric analyses are presented in Figure 3A,B. Protein expression levels were normalized to GAPDH, which served as the internal reference protein. In kidney tissue, Bax protein expression in the 12 mg/kg CP group (*p* = 0.027) increased approximately 8.6-fold compared to the control group, whereas Bcl-2 expression decreased by 80% (*p* = 0.002). In heart tissue, Bax levels in the 12 mg/kg CP group (*p* = 0.017) showed a 2.8-fold increase, accompanied by a 79% reduction in Bcl-2 expression (*p* = 0.022).

### 3.4. Histopathological and Immunohistochemical Evaluation of CP-Induced Tissue Effects

**Figure 4:**
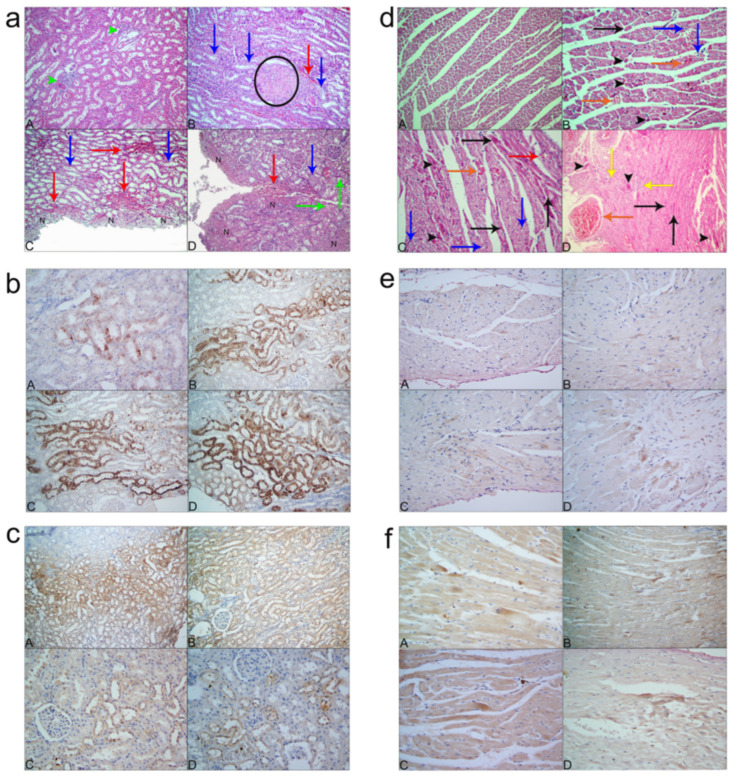
Histopathological and immunohistochemical analyses of kidney and heart tissues in a dose-dependent manner following CP administration. (**a**) (**A**) **Control group:** Focal MCI in the interstitial area (green arrowheads), ×20, H&E. (**B**) **CP 5 mg/kg group:** Focal necrotic area and MCI around the necrotic region (black circle), hydropic degeneration in tubular epithelial cells (blue arrows), and hemorrhagic areas in the renal parenchyma (red arrow), ×20, H&E. (**C**) **CP 7.5 mg/kg group:** Extensive necrotic area in the subcapsular region (indicated by “N”), hemorrhagic areas around the necrotic region and within the parenchyma (red arrows), and hydropic degeneration in tubular epithelial cells (blue arrows), ×20, H&E. (**D**) **CP 12 mg/kg group:** Large necrotic areas extending from the subcapsular region into the parenchymal tissue and MCI around the necrotic area (indicated by “N”), hemorrhagic regions around necrosis (red arrows), hydropic degeneration in tubular epithelial cells (blue arrows), and hyaline casts within tubular lumens (green arrows), ×20, H&E. (**b**) Bax immunoreactivity in renal tubular epithelial cells (IHC). (**A**) Control group, ×40. (**B**) CP 5 mg/kg group, ×20. (**C**) CP 7.5 mg/kg group, ×20. (**D**) CP 12 mg/kg group, ×20. (**c**) Bcl-2 immunoreactivity in renal tubular epithelial cells (IHC). (**A**) Control group, ×40. (**B**) CP 5 mg/kg group, ×20. (**C**) CP 7.5 mg/kg group, ×20. (**D**) CP 12 mg/kg group, ×20. (**d**) (**A**) **Control group:** General appearance of cardiac tissue, ×20, H&E. (**B**) **CP 5 mg/kg group:** Cardiomyocytes with pyknotic nuclei (black arrowheads), necrosis (black arrows) and hydropic degeneration (blue arrows) in cardiomyocytes, and vascular congestion (orange arrows), ×40, H&E. (**C**) **CP 7.5 mg/kg group:** Cardiomyocytes with pyknotic nuclei (black arrowheads), necrosis (black arrows) and hydropic degeneration (blue arrows) in cardiomyocytes, and vascular congestion (orange arrow), focal myocardial hemorrhage (red arrow) ×40, H&E. (**D**) **CP 12 mg/kg group**: Cardiomyocytes with pyknotic nuclei (black arrowheads), necrosis (black arrows), focal necrotic foci and MCI in the parenchyma (yellow arrows), and vascular congestion (orange arrow), ×20, H&E. (**e**) Bax immunoreactivity in cardiomyocytes (IHC, ×40). (**A**) Control group, ×40. (**B**) CP 5 mg/kg group. (**C**) CP 7.5 mg/kg group. (**D**) CP 12 mg/kg group. (**f**) Bcl-2 immunoreactivity in cardiomyocytes (IHC). (**A**) Control group, ×40. (**B**) CP 5 mg/kg group, ×20. (**C**) CP 7.5 mg/kg group, ×40. (**D**) CP 12 mg/kg group, ×40.

The histopathological scoring results and corresponding statistical analyses of kidney and heart tissues obtained from rats are presented in Table 1 and Table 2, respectively. Histopathological examination of kidney tissues revealed a dose-related increase in hydropic degeneration, necrosis (particularly in the epithelial cells of the collecting ducts), and hyaline cast formation (within tubular and collecting duct lumina) with increasing doses of CP. In contrast, parameters such as tubular dilatation, glomerular atrophy, IMCI, perivascular edema, and hemorrhage showed statistically significant differences when compared to the control group; however, no significant variations were observed among the dose groups. Additionally, interstitial edema and congestion parameters exhibited no significant differences between groups (Figure 4a).

Histopathological examination of the heart tissues revealed a dose-related increase in hydropic degeneration, focal necrosis, and MCI with increasing doses of CP. Although congestion and hemorrhage parameters differed from those of the control group, no statistically significant differences were observed among the CP dose groups. The level of interstitial edema showed a significant difference compared to the control group; however, no variation was detected between the dose groups (Figure 4d). In addition to these findings, separation of muscle fibers was noted, along with swelling in vascular endothelial cells. Amyloid deposits were observed in the walls of certain blood vessels and within the myocardial parenchyma. As the CP dose increased, mucopolysaccharide accumulation and myxomatous changes characterized by loose connective tissue were detected in the cardiac valves.

In this study, immunohistochemical staining for Bax and Bcl-2 proteins was performed on sections obtained from kidney and heart tissues. The statistical analyses of the scoring data are presented in Table 3. In kidney sections, a dose-dependent increase in Bax immunoreactivity was observed. Specifically, the 5, 7.5, and 12 mg/kg CP groups showed statistically significant differences in the intensity and extent of positive staining compared with the control group. While no significant difference was detected between the 5 mg/kg and 7.5 mg/kg CP groups, a significant increase in both staining intensity and extent was observed in the 12 mg/kg CP group relative to the other groups. In contrast, Bcl-2 immunostaining progressively decreased with increasing CP dose, showing significant differences among all groups. When all groups were compared in terms of staining intensity and extent, statistically significant differences were detected between each group (Figure 4b,c). In heart sections, no statistically significant differences were found among the groups in terms of Bax or Bcl-2 immunoreactivity (Figure 4e,f).

## 4. Discussion

The present study provides an integrated early comparative evaluation of cisplatin-induced renal and cardiac toxicity within the same acute experimental framework using combined biochemical, oxidative stress, histopathological, immunohistochemical, and apoptotic analyses. CP is an effective antineoplastic agent composed of platinum coordinated with chloride and ammonia ligands and is extensively used in the treatment of a variety of tumors [6,25]. However, among its numerous complications, nephrotoxicity remains the most prominent [26,27]. The antitumor activity and nephrotoxic effects of CP are primarily attributed to the formation of platinum–DNA adducts following intracellular activation of cisplatin, leading to DNA damage, cell cycle arrest, and apoptosis [6,25,27]. In addition to inducing apoptosis, CP also triggers necrosis. However, necrosis typically occurs at higher doses, where mitochondrial injury is more pronounced, whereas apoptosis predominates at therapeutic concentrations, where cellular damage is relatively limited [12,28,29,30]. Although the precise mechanism by which CP induces nephrotoxicity remains unclear, it has been demonstrated that in adult individuals, the proximal tubular cells, which do not undergo division [7,11,27], are particularly susceptible to injury. This suggests that the damage is primarily caused not by DNA lesions but rather by oxidative stress and inflammation [7,27,31]. The observed findings suggest progressive dose-related acute toxic responses, with more pronounced alterations observed at higher cisplatin doses.

In in vivo studies, both apoptotic and necrotic pathways have been reported to be activated following CP exposure [27,31,32]. Although multiple mechanisms, including endoplasmic reticulum stress and mitochondrial apoptotic pathways, have been implicated in CP-induced nephrotoxicity, their relative contributions to overall renal injury have not been fully elucidated [11,12,28]. According to the histopathological findings of the present study, increasing CP doses in kidney tissue resulted in elevated levels of hydropic degeneration, necrosis, IMCI, tubular dilatation, and glomerular atrophy, whereas interstitial and perivascular edema, congestion, and hemorrhage parameters remained unchanged. Although TNF-α levels increased at the highest cisplatin dose, IL-6, IL-10, and IL-1β did not demonstrate a strong progressive dose-related pattern within the acute 3-day observation period. Therefore, oxidative stress-related alterations appeared to be more prominent than cytokine-mediated inflammatory activation during this early phase. The observed increase in necrosis is consistent with previous studies reporting that necrotic cell death occurs predominantly at higher CP doses [12,31,32]. Moreover, since apoptosis is known to be induced by oxidative stress and inflammation, various antioxidant agents have been investigated in previous studies for their potential to mitigate CP-induced nephrotoxicity. In the present study, it was observed that with increasing CP doses, the levels of MDA and LPO increased, whereas SOD levels decreased.

CP-induced nephrotoxicity has been associated with oxidative stress, inflammation, and activation of apoptotic pathways [11,27,33,34]. Consistent with these findings, our findings indicate increases in selected inflammatory markers, particularly TNF-α at the highest dose. Previous studies have reported that several inflammatory mediators, including IL-1β and IL-6, may contribute to CP-induced inflammatory signaling [11,31,35]. Moreover, NF-κB plays a central role in regulating the expression of these pro-inflammatory cytokines [8]. Similarly, in the study by Al Za’abi et al., different doses of CP were shown to cause dose-dependent increases in TNF-α and IL-1β in the kidneys, as well as elevations in IL-18 and L-FABP levels in the urine, accompanied by significant rises in renal homogenate inflammation indices [36]. Furthermore, it has been reported that renal transcription factor Nrf2 and renal caspase-3 levels increase in a dose-dependent manner. However, except for TNF-α elevation at the highest cisplatin dose, IL-6, IL-10, and IL-1β did not demonstrate strong progressive dose-related activation within the acute 3-day observation period. Therefore, oxidative stress-related alterations appeared to be more prominent than cytokine-mediated inflammatory responses during this early phase.

CP has been shown to trigger apoptosis in renal tubular epithelial cells through multiple mechanisms, including the endoplasmic reticulum (ER) stress pathway and the activation of pro-apoptotic proteins [37,38]. Additionally, CP-induced nephrotoxicity has been shown to be attenuated in Bax-deficient mice, highlighting the critical role of Bax-mediated apoptosis in renal injury [13]. In the present study, the apoptotic pathway was evaluated across increasing dose levels both at the protein level and through immunohistochemical analysis. According to our results, the levels of apoptotic proteins changed significantly compared to the control group. In kidney tissue, although Bax levels in the 12 mg/kg and 7.5 mg/kg groups were relatively similar, both showed a significant increase compared to the control and the lowest-dose groups.

A similar pattern was observed for Bcl-2, where its expression decreased in a dose-dependent manner. These findings are consistent with previously published data describing CP-induced activation of apoptotic pathways. The immunohistochemical analyses further supported the biochemical results. In kidney tissues, Bax expression showed comparable staining intensity in the 5 mg/kg and 7.5 mg/kg groups, whereas a significant increase was observed in the 12 mg/kg group. Conversely, Bcl-2 expression exhibited a progressive and statistically significant decrease across all dose groups as the CP concentration increased. Cardiovascular toxicity has increasingly been recognized as a clinically relevant complication of platinum-based chemotherapy. [2]. Although less frequently studied than nephrotoxicity, cardiovascular complications associated with cisplatin exposure have also been reported, including vascular dysfunction, myocardial injury, and an increased risk of cardiovascular events [2,39,40]. Despite the absence of a precise definition for CP-related cardiotoxicity, several studies have demonstrated that CP can induce inflammation, oxidative stress, mitochondrial dysfunction, and activation of apoptotic pathways in cardiac tissue [39,40,41]. Increased ROS generation and impairment of antioxidant defense systems have been recognized as major contributors to CP-induced cardiac injury and oxidative stress. Due to the low levels of antioxidant enzymes, cardiac tissue becomes particularly susceptible to oxidative stress [39,40]. Western blot analysis evaluates protein expression in homogenized tissue extracts representing broader myocardial regions, whereas immunohistochemistry reflects localized spatial protein distribution within thin tissue sections. In addition, Western blotting generally provides higher analytical sensitivity and quantitative dynamic range compared with chromogenic immunohistochemical staining methods. Furthermore, formalin fixation and paraffin embedding may contribute to epitope masking in cardiac tissue, potentially reducing immunohistochemical sensitivity.

In CP-exposed cardiomyocytes, oxidative stress, mitochondrial dysfunction, and apoptotic signaling have been reported [39,40,41]. Moreover, alterations in apoptotic proteins and genes may substantially influence the survival of cardiomyocytes following CP exposure [40,41]. In the present study, similar to the findings in renal tissue, Bax levels in cardiac tissue showed comparable results between the 12 mg/kg and 7.5 mg/kg groups, yet both were significantly increased compared to the control and the lowest-dose groups. Although Bcl-2 levels in the dose groups differed significantly from the control group, no significant difference was observed among the dose groups themselves. According to the results of the immunohistochemical analyses, although variations in Bax and Bcl-2 expression levels were observed in cardiac tissue both when compared with the control group and among the dose groups—these changes were not statistically significant. The discrepancy between Western blot and immunohistochemical findings suggests a method-dependent detection sensitivity, indicating that protein-level alterations may not always be reflected at the tissue distribution level.

Based on the histopathological findings, the marked increase in necrotic histopathological findings within cardiac tissue may suggest a substantial contribution of necrotic injury, particularly at higher CP doses. This is consistent with previous reports indicating that necrosis occurs at higher doses of CP due to severe mitochondrial damage, whereas apoptosis predominates at therapeutic concentrations with relatively limited cellular injury [12,28,30]. The results obtained in this study may be attributed to the acute administration of CP. It is plausible that chronic administration could reveal more pronounced or distinct effects. Indeed, Herradón et al. demonstrated that chronic CP treatment leads to dose-dependent cardiovascular alterations [2]. At lower CP doses, vascular toxicity and cardiac or systemic cardiovascular changes have been reported, whereas at higher doses, these vascular toxic effects persist and are accompanied by blood pressure alterations. It has been reported that at the highest CP dose, there is a significant decrease in diastolic blood pressure accompanied by mild bradycardia, whereas systolic blood pressure remains unchanged after treatment. Additionally, low and moderate doses of CP have been shown to exert no significant effect on heart rate [2]. In the present study, histopathological analysis of cardiac tissue revealed a dose-dependent increase in hydropic degeneration, necrosis, interstitial edema, congestion, hemorrhage, and MCI. These findings are consistent with previously published data, further supporting the association between CP exposure and dose-related cardiac tissue damage. In the literature, both acute administration of CP (10 mg/kg) and chronic treatment have been described as inducing cytotoxic effects characterized by disruption of myofibrils, separation of cardiac muscle fibers, and structural alterations in heart tissue, including interstitial hemorrhage and fibrosis [2,39,40]. The same studies also reported a significant increase in caspase-3 activity, nuclear DNA fragmentation, and a reduction in cardiomyocyte cross-sectional area in cardiac tissue. These findings likely reflect the contribution of apoptotic cell death to the observed cardiac damage [40]. Topal et al. reported polymorphonuclear leukocyte infiltration, dilated congested blood vessels, hemorrhage, edema, necrosis, and pyknotic nuclei in the cardiac tissues of CP-treated rats [5]. The edema observed in cardiac tissue may be interpreted as a characteristic feature of inflammatory reactions associated with cell injury and death, as described in Robbins Pathologic Basis of Disease [42]. In another study, El-Awady et al. showed that increasing CP doses altered the structural integrity of cardiac tissue, resulting in wavy cardiomyocytes and disorganization of myofibrils [39]. Following cisplatin exposure, rats exhibited impaired myocardial contractile function and cardiac injury [41]. These alterations may be attributed to mitochondrial dysfunction, oxidative stress, and enhanced apoptosis [39,40,41]. Furthermore, El-Sawalhi and Ahmed demonstrated that administration of CP (7 mg/kg) in rats resulted in a marked loss of mitochondrial transmembrane potential, a significant increase in myocardial caspase-3 levels, a notable reduction in intact genomic DNA, and a decrease in cardiomyocyte cross-sectional area, collectively indicating substantial mitochondrial and apoptotic injury in cardiac tissue. The increase in ROS induced by CP has been shown to disrupt the mitochondrial transmembrane potential, which serves as an indicator of cellular energy status and homeostasis in cardiac tissue [40]. This disruption facilitates the release of apoptotic cytochrome c from mitochondria, thereby triggering mitochondria-dependent apoptosis [10,33,34,38].

Consequently, CP exposure contributes to enhanced mitochondrial injury [40,41]. Thus, the loss of mitochondrial transmembrane potential may serve as a marker of mitochondrial dysfunction. Previous studies have also described mitochondrial impairment as a contributing factor in CP-induced nephrotoxicity [29,43]. In the literature, CP-induced cardiac injury has been associated with bradycardia, ventricular dysfunction, and reductions in heart rate and blood pressure [2,39].

These alterations are thought to be linked to mitochondrial abnormalities, increased oxidative stress, and apoptotic stimulation [40,41]. In another study, CP administration in cardiac tissue was shown to cause a significant increase in thiobarbituric acid reactive substances levels, accompanied by a marked decrease in GSH levels and reduced SOD, GPx, and catalase enzyme activities [5]. Similarly, in the present study, increasing CP doses led to elevated MDA and LPO levels and decreased SOD activity in cardiac tissue. This effect, as also suggested by the current findings, may result in enhanced lipid peroxidation of cardiac membranes, leading to structural alterations in membrane integrity and tissue damage, thereby causing the leakage of intracellular enzymes into the extracellular space [39,40,44]. In the study by Topal et al., CP-treated rat cardiac tissues showed a significant increase in MDA levels accompanied by a significant decrease in tGSH levels, as well as elevated concentrations of the inflammatory cytokines IL-1β and TNF-α [5]. Previous studies have reported that both IL-1β and TNF-α can contribute to systemic tissue damage [35,45]. Consistent with these findings, the present study demonstrated dose-related increases primarily in TNF-α levels, while other inflammatory cytokines showed limited or non-progressive changes across dose groups. IL-1β plays a crucial role in the inflammatory cascade by promoting apoptosis and leukocyte infiltration [35]. TNF-α and IL-1β are known to appear during the early phase of inflammation, where they mediate several key functions such as neutrophil oxidative burst and release of ROS through shared signaling pathways [35,45]. Interestingly, not all oxidative stress markers showed a uniform response, as 4-HNE levels remained unchanged, which may reflect marker-specific sensitivity or differences in the temporal dynamics of lipid peroxidation. The findings may be relevant to human biology because CP-induced oxidative stress, inflammation, and apoptosis-related pathways are biologically conserved mechanisms involved in organ toxicity. However, generalisation to humans or other species should be made cautiously because this study used an acute rat model, a single post-treatment time point, and experimentally administered cisplatin doses. Therefore, the results are best interpreted as early mechanistic evidence of dose-related cardiorenal toxicity rather than direct clinical evidence. Recent advances in nanotechnology-assisted cancer therapies and cancer vaccine strategies may contribute to reducing systemic toxicities associated with conventional chemotherapeutic agents such as CP by improving targeted drug delivery and minimizing off-target tissue injury [46].

## 5. Conclusions

In conclusion, acute CP exposure produced dose-related biochemical, molecular, and histopathological alterations in cardiac and renal tissues, with evidence of tissue-specific sensitivity. More pronounced injury patterns were observed at higher CP doses. The findings support the importance of dose selection in experimental modeling and provide a mechanistic basis for future studies investigating chronic and cumulative toxicity.

### Limitations

This study has several limitations, including a relatively small sample size and the use of an acute CP administration model. Although this design enabled the evaluation of early toxic effects, it does not allow conclusions to be drawn regarding long-term or progressive tissue injury. Chronic exposure models may therefore offer additional insight into the sustained and cumulative effects of CP. Accordingly, the present study should be regarded as exploratory, and its findings should be interpreted within the context of these limitations. Given the acute design of the present study, the findings should be interpreted as early-phase responses. Future studies incorporating chronic exposure models are warranted to determine whether these early-phase responses translate into long-term tissue injury. Such approaches may also help clarify tissue-specific susceptibility and cumulative toxicity patterns. Additional limitations of the present study include the absence of temporal multi-point kinetic analyses, the lack of highly sensitive early renal injury biomarkers such as KIM-1 and NGAL, and the absence of caspase pathway analyses. Furthermore, fluorescent apoptosis staining methods such as Calcein AM, ethidium homodimer, or Hoechst staining were not included in the experimental design. Therefore, the apoptotic findings should be interpreted primarily based on Bax/Bcl-2 protein alterations together with histopathological and immunohistochemical observations. Future studies incorporating detailed apoptotic pathway analyses and advanced cell death imaging techniques may provide additional mechanistic insight into cisplatin-induced cardiorenal toxicity. In addition, the acute single-dose experimental design may not fully reflect the cumulative and chronic toxic effects observed during clinical CP chemotherapy protocols.

## Figures and Tables

**Table 1:** Kidney Histopathological Scores.

Kidney Histopathological Analyses										
Groups	Hydropic Degeneration	Necrosis	Tubular Dilatation	Glomerular Atrophy	Hyaline Cylinder	MNC	Interstitial Edema	Perivascular Edema	Congestion	Bleeding
Control	0.42 ± 0.15 ^a^	0.50 ± 0.29 ^a^	0.58 ± 0.29 ^a^	0.33 ± 0.17 ^a^	0.91 ± 0.30 ^a^	0.58 ± 0.24 ^a^	0.33 ± 0.16 ^a^	0.16 ± 0.11 ^a^	0.75 ± 0.21 ^a^	0.41 ± 0.23 ^a^
5 mg/kg CP	1.33 ± 0.17 ^b^	1.41 ± 0.20 ^b^	0.92 ± 0.15 ^ab^	0.58 ± 0.15 ^ab^	1.58 ± 0.15 ^a^	1.33 ± 0.17 ^b^	0.41 ± 0.15 ^a^	0.25 ± 0.17 ^a^	1.33 ± 0.17 ^a^	1.33 ± 0.26 ^b^
7.5 mg/kg CP	1.67 ± 0.17 ^bc^	1.50 ± 0.18 ^b^	1.25 ± 0.17 ^b^	1.25 ± 0.25 ^bc^	1.67 ± 0.36 ^ab^	1.33 ± 0.17 ^b^	0.67 ± 0.26 ^a^	0.58 ± 0.15 ^ab^	1.00 ± 0.13 ^a^	1.58 ± 0.15 ^b^
12 mg/kg CP	2.33 ± 0.40 ^c^	2.25 ± 0.25 ^c^	1.42 ± 0.24 ^b^	1.58 ± 0.40 ^c^	2.66 ± 0.49 ^b^	1.58 ± 0.33 ^b^	0.75 ± 0.11 ^a^	0.83 ± 0.17 ^b^	1.41 ± 0.35 ^a^	2.00 ± 0.22 ^b^

Different letters in the same column are statistically significant according to one-way ANOVA and post hoc Tukey’s HSD test. CP: Cisplatin.

**Table 2:** Heart Histopathological Scores.

Heart Histopathological Analyses						
Groups	Hydropic Degeneration	Necrosis	Interstitial Edema	Congestion	Bleeding	MNC
Control	0.66 ± 0.11 ^a^	0.66 ± 0.17 ^a^	0.33 ± 0.17 ^a^	0.66 ± 0.17 ^a^	0.58 ± 0.20 ^a^	0.41 ± 0.15 ^a^
5 mg/kg CP	1.25 ± 0.21 ^b^	1.25 ± 0.25 ^a^	0.92 ± 0.15 ^b^	1.00 ± 0.13 ^a^	0.75 ± 0.11 ^a^	1.00 ± 0.13 ^b^
7.5 mg/kg CP	1.67 ± 1.17 ^b^	1.91 ± 0.27 ^b^	0.83 ± 0.17 ^b^	1.66 ± 0.17 ^b^	1.91 ± 0.15 ^b^	1.16 ± 0.21 ^b^
12 mg/kg CP	2.58 ± 0.15 ^c^	2.67 ± 0.17 ^c^	1.33 ± 0.17 ^b^	2.41 ± 0.27 ^b^	2.33 ± 0.21 ^b^	2.16 ± 0.21 ^c^

Different letters in the same column are statistically significant according to one-way ANOVA and post hoc Tukey’s HSD test. CP: Cisplatin.

**Table 3:** Immunohistochemical scoring of kidney and heart tissues.

Groups	Kidney		Heart		Kidney	Heart
	Bax	Bcl-2	Bax	Bcl-2	Bax/Bcl-2	
Control	1.17 ± 0.17 ^a^	4.50 ± 0.5 ^d^	1.50 ± 0.34 ^a^	2.66 ± 0.33 ^a^	0.28 ± 0.05 ^a^	0.62 ± 0.18 ^a^
5 mg/kg CP	2.33 ± 0.42 ^b^	3.33 ± 0.21 ^c^	2.00 ± 0.45 ^a^	2.50 ± 0.43 ^a^	0.71 ± 0.14 ^a^	0.91 ± 0.24 ^a^
7.5 mg/kg CP	3.00 ± 0.37 ^b^	2.33 ± 0.21 ^b^	2.08 ± 0.27 ^a^	2.16 ± 0.31 ^a^	1.30 ± 0.16 ^a^	1.09 ± 0.22 ^a^
12 mg/kg CP	4.66 ± 0.42 ^c^	1.25 ± 0.17 ^a^	2.50 ± 0.43 ^a^	2.08 ± 0.20 ^a^	4.11 ± 0.68 ^b^	1.30 ± 0.21 ^a^

Different letters in the same column are statistically significant according to one-way ANOVA and post hoc Tukey’s HSD test. CP: Cisplatin.

## Data Availability

Data will be made available on request.

## Abbreviations

CP	Cisplatin
ELISA	Enzyme-Linked Immunosorbent Assay
MDA	Malondialdehyde
SOD	Superoxide Dismutase
LPO	Lipid peroxidase
ER	endoplasmic reticulum
AIF	Apoptosis-inducing factor
i.p.	Intraperitoneal
RIPA	Radioimmunoprecipitation Assay
BCA	bicinchoninic acid
BSA	Bovine serum albumin
4-HNE	4-hydroxy-2-nonenal
IL-6	Interleukin-6
IL-10	Interleukin-10
IL-1β	Interleukin-1β
TNF-α	Tumor necrosis factor
CK-MB	Creatine kinase-MB
PVDF	Polyvinylidene Fluoride
RT	Room temperature
H&E	Hematoxylin eosin
IMCI	interstitial mononuclear cell infiltrate
MCI	mononuclear cell infiltrate
IHC	immunohistochemical
HIER 1	Heat-induced epitope retrieval
DAB	3,3′-diaminobenzidine
ROS	Reactive oxygen species
NF-κB	Nuclear factor-κB
L-FABP	Liver Fatty Acid-Binding Protein
Nrf2	Nuclear factor erythroid-related factor 2
EPO	erythropoietin
DNA	Deoxyribonucleic acid
GSH	Glutathione

## References

[1] Chirino Y.I., Hernández-Pando R., Pedraza-Chaverrí J. (2004). Peroxynitrite decomposition catalyst ameliorates renal damage and protein nitration in cisplatin-induced nephrotoxicity in rats. BMC Pharmacol..

[2] Herradón E., González C., Uranga J.A., Abalo R., Martín M.I., López-Miranda V. (2017). Characterization of Cardiovascular Alterations Induced by Different Chronic Cisplatin Treatments. Front. Pharmacol..

[3] Mollman J.E. (1990). Cisplatin neurotoxicity. N. Engl. J. Med..

[4] Yousef M.I., Saad A.A., El-Shennawy L.K. (2009). Protective effect of grape seed proanthocyanidin extract against oxidative stress induced by cisplatin in rats. Food Chem. Toxicol..

[5] Topal İ., Özbek Bilgin A., Keskin Çimen F., Kurt N., Süleyman Z., Bilgin Y., Özçiçek A., Altuner D. (2018). The effect of rutin on cisplatin-induced oxidative cardiac damage in rats. Anatol. J. Cardiol..

[6] Fuertes M.A., Castilla J., Alonso C., Pérez J.M. (2003). Cisplatin biochemical mechanism of action: From cytotoxicity to induction of cell death through interconnections between apoptotic and necrotic pathways. Curr. Med. Chem..

[7] Yao X., Panichpisal K., Kurtzman N., Nugent K. (2007). Cisplatin nephrotoxicity: A review. Am. J. Med. Sci..

[8] dos Santos N.A., Rodrigues M.A.C., Martins N.M., dos Santos A.C. (2012). Cisplatin-induced nephrotoxicity and targets of nephroprotection: An update. Arch. Toxicol..

[9] Miller R.P., Tadagavadi R.K., Ramesh G., Reeves W.B. (2010). Mechanisms of Cisplatin nephrotoxicity. Toxins.

[10] Lee R.H., Song J.M., Park M.Y., Kang S.K., Kim Y.K., Jung J.S. (2001). Cisplatin-induced apoptosis by translocation of endogenous Bax in mouse collecting duct cells. Biochem. Pharmacol..

[11] Ozkok A., Edelstein C.L. (2014). Pathophysiology of cisplatin-induced acute kidney injury. Biomed. Res. Int..

[12] Lieberthal W., Triaca V., Levine J. (1996). Mechanisms of death induced by cisplatin in proximal tubular epithelial cells: Apoptosis vs. necrosis. Am. J. Physiol..

[13] Wei Q., Dong G., Franklin J., Dong Z. (2007). The pathological role of Bax in cisplatin nephrotoxicity. Kidney Int..

[14] Yin X., Apostolov E.O., Shah S.V., Wang X., Bogdanov K.V., Buzder T., Stewart A.G., Basnakian A.G. (2007). Induction of renal endonuclease G by cisplatin is reduced in DNase I-deficient mice. J. Am. Soc. Nephrol..

[15] Kaushal G.P., Kaushal V., Hong X., Shah S.V. (2001). Role and regulation of activation of caspases in cisplatin-induced injury to renal tubular epithelial cells. Kidney Int..

[16] Kumburovic I., Selakovic D., Juric T., Jovicic N., Mihailovic V., Stankovic J.K., Sreckovic N., Kumburovic D., Jakovljevic V., Rosic G. (2019). Antioxidant Effects of *Satureja hortensis* L. Attenuate the Anxiogenic Effect of Cisplatin in Rats. Oxid. Med. Cell. Longev..

[17] Erdem T., Bayindir T., Filiz A., Iraz M., Selimoglu E. (2012). The effect of resveratrol on the prevention of cisplatin ototoxicity. Eur. Arch. Otorhinolaryngol..

[18] Karavelioglu E., Boyaci M.G., Simsek N., Sonmez M.A., Koc R., Karademir M., Guven M., Eser O. (2015). Selenium protects cerebral cells by cisplatin induced neurotoxicity. Acta Cir. Bras..

[19] Luna L.G. (1968). Manual of Histologic Staining Methods of the Armed Forces Institute of Pathology.

[20] Dik B., Hatipoglu D., Ates M.B. (2024). Potential effects of Resatorvid and alpha lipoic acid on gentamicin-induced nephrotoxicity in rats. Pharmacol. Res. Persp..

[21] Kavakli K., Hatipoglu D., Bulut A., Ates M.B. (2025). Protective effect of ursodeoxycholic acid in a co-exposure model of cadmium-induced kidney damage. Toxicol. Ind. Health.

[22] Türkmen N.B., Özek D.A., Taşlıdere A., Çiftçi O., Saral O., Gül C.C. (2022). Protective Role of *Diospyros lotus* L. in Cisplatin-Induced Cardiotoxicity: Cardiac Damage and Oxidative Stress in Rats. Turk. J. Pharm. Sci..

[23] Saleh R.M., Awadin W.F., Elseady Y.Y., Waheish F. (2014). Renal and cardiovascular damage induced by cisplatin in rats. Life Sci. J..

[24] Hameed K.A., Banumathi A., Ulaganathan G. (2015). Performance evaluation of maximal separation techniques in immunohistochemical scoring of tissue images. Micron.

[25] Wang D., Lippard S.J. (2005). Cellular processing of platinum anticancer drugs. Nat. Rev. Drug Discov..

[26] Ali B.H., Al Moundhri M.S. (2006). Agents ameliorating or augmenting the nephrotoxicity of cisplatin and other platinum compounds: A review of some recent research. Food Chem. Toxicol..

[27] Pabla N., Dong Z. (2008). Cisplatin nephrotoxicity: Mechanisms and renoprotective strategies. Kidney Int..

[28] Bonegio R., Lieberthal W. (2002). Role of apoptosis in the pathogenesis of acute renal failure. Curr. Opin. Nephrol. Hypertens..

[29] Zsengellér Z.K., Ellezian L., Brown D., Horváth B., Mukhopadhyay P., Kalyanaraman B., Parikh S.M., Karumanchi S.A., Stillman I.E., Pacher P. (2012). Cisplatin nephrotoxicity involves mitochondrial injury with impaired tubular mitochondrial enzyme activity. J. Histochem. Cytochem..

[30] Kim J.S., He L., Lemasters J.J. (2003). Mitochondrial permeability transition: A common pathway to necrosis and apoptosis. Biochem. Biophys. Res. Commun..

[31] Ramesh G., Reeves W.B. (2003). TNFR2-mediated apoptosis and necrosis in cisplatin-induced acute renal failure. Am. J. Physiol. Ren. Physiol..

[32] Faubel S., Ljubanovic D., Reznikov L., Somerset H., Dinarello C.A., Edelstein C.L. (2004). Caspase-1-deficient mice are protected against cisplatin-induced apoptosis and acute tubular necrosis. Kidney Int..

[33] Seth R., Yang C., Kaushal V., Shah S.V., Kaushal G.P. (2005). p53-dependent caspase-2 activation in mitochondrial release of apoptosis-inducing factor and its role in renal tubular epithelial cell injury. J. Biol. Chem..

[34] Jiang M., Wei Q., Wang J., Du Q., Yu J., Zhang L., Dong Z. (2006). Regulation of PUMA-alpha by p53 in cisplatin-induced renal cell apoptosis. Oncogene.

[35] Dinarello C.A. (2000). Proinflammatory cytokines. Chest.

[36] Al Za’abi M., Al Salam S., Al Suleimani Y., Ashique M., Manoj P., Nemmar A., Ali B.H. (2021). Effects of repeated increasing doses of cisplatin as models of acute kidney injury and chronic kidney disease in rats. Naunyn Schmiedebergs Arch. Pharmacol..

[37] Liu H., Baliga R. (2005). Endoplasmic reticulum stress-associated caspase 12 mediates cisplatin-induced LLC-PK1 cell apoptosis. J. Am. Soc. Nephrol..

[38] Park M.S., De Leon M., Devarajan P. (2002). Cisplatin induces apoptosis in LLC-PK1 cells via activation of mitochondrial pathways. J. Am. Soc. Nephrol..

[39] El-Awady E.S.E., Moustafa Y.M., Abo-Elmatty D.M., Radwan A. (2011). Cisplatin-induced cardiotoxicity: Mechanisms and cardioprotective strategies. Eur. J. Pharmacol..

[40] El-Sawalhi M.M., Ahmed L.A. (2014). Exploring the protective role of apocynin, a specific NADPH oxidase inhibitor, in cisplatin-induced cardiotoxicity in rats. Chem. Biol. Interact..

[41] Ma H., Jones K.R., Guo R., Xu P., Shen Y., Ren J. (2010). Cisplatin compromises myocardial contractile function and mitochondrial ultrastructure: Role of endoplasmic reticulum stress. Clin. Exp. Pharmacol. Physiol..

[42] Cotran R.S., Kumar V., Collins T. (1999). Robbins Pathologic Basis of Disease.

[43] Santos N.A., Catão C.S., Martins N.M., Curti C., Bianchi M.L.P., Santos A.C. (2007). Cisplatin-induced nephrotoxicity is associated with oxidative stress, redox state unbalance, impairment of energetic metabolism and apoptosis in rat kidney mitochondria. Arch. Toxicol..

[44] Rjiba-Touati K., Ayed-Boussema I., Belarbia A., Achour A., Bacha H. (2012). Recombinant human erythropoietin prevents cisplatin-induced genotoxicity in rat liver and heart tissues via an antioxidant process. Drug Chem. Toxicol..

[45] Semenzato G. (1990). Tumour necrosis factor: A cytokine with multiple biological activities. Br. J. Cancer..

[46] Udayakumar S., Pandiarajan S., Mercy D.J., Suresh J., Kumar J.R.J., Girigoswami A., Girigoswami K. (2025). Revolutionizing Cancer Vaccine: The Power of Advanced Nanotechnology. Chemistry.

